# Chrysophanic acid reduces testosterone-induced benign prostatic hyperplasia in rats by suppressing 5α-reductase and extracellular signal-regulated kinase

**DOI:** 10.18632/oncotarget.13430

**Published:** 2016-11-17

**Authors:** Dong-Hyun Youn, Jinbong Park, Hye-Lin Kim, Yunu Jung, JongWook Kang, Mi-Young Jeong, Gautam Sethi, Kwang Seok Ahn, Jae-Young Um

**Affiliations:** ^1^ Department of Science in Korean Medicine, Graduate School, Kyung Hee University, Dongdaemun-Gu, Seoul, 02447, Republic of Korea; ^2^ College of Korean Medicine, Basic Research Laboratory for Comorbidity Regulation, Kyung Hee University, Dongdaemun-Gu, Seoul, 02447, Republic of Korea; ^3^ Department of Pharmacology, Yong Loo Lin School of Medicine, National University of Singapore, 117600, Singapore

**Keywords:** benign prostatic hyperplasia-BPH, chrysophanic acid, prostate specific antigen, 5α-reductase, extracellular signal-regulated kinase

## Abstract

Benign prostatic hyperplasia (BPH) is one of the most common chronic diseases in male population, of which incidence increases gradually with age. In this study, we investigated the effect of chrysophanic acid (CA) on BPH. BPH was induced by a 4-week injection of testosterone propionate (TP). Four weeks of further injection with vehicle, TP, TP + CA, TP + finasteride was carried on. In the CA treatment group, the prostate weight was reduced and the TP-induced histological changes were restored as the normal control group. CA treatment suppressed the TP-elevated prostate specific antigen (PSA) expression. In addition, 5α-reductase, a crucial factor in BPH development, was suppressed to the normal level close to the control group by CA treatment. The elevated expressions of androgen receptor (AR), estrogen receptor α and steroid receptor coactivator 1 by TP administration were also inhibited in the CA group when compared to the TP-induced BPH group. Then we evaluated the changes in three major factors of the mitogen-activated protein kinase chain during prostatic hyperplasia; extracellular signal-regulated kinase (ERK), c-Jun-N-terminal kinase (JNK) and p38 mitogen-activated protein kinase (p38). While ERK was elevated in the process of BPH, JNK and p38 was not changed. This up-regulated ERK was also reduced as normal by CA treatment. Further *in vitro* studies with RWPE-1 cells confirmed TP-induced proliferation and elevated AR, PSA and p-ERK were all reduced by CA treatment. Overall, these results suggest a potential pharmaceutical feature of CA in the treatment of BPH.

## INTRODUCTION

Benign prostatic hyperplasia (BPH) is an extremely common disease in aging men, originally meaning hyperplasia of the prostate gland, an encapsulated accessory sex gland that surrounds the bladder neck and proximal urethra. The term BPH stands for the histological diagnosis of unregulated proliferation in the prostate tissue, however, the recent main focus of BPH moves from the justification of BPH on to the major symptoms of it, which can negatively impact the quality of the patient's life. The clinical symptoms of BPH are urgency, frequency, weak stream, nocturia and incomplete emptying of urine, which are also known as lower urinary tract symptoms (LUTS) [1]. Casabe and colleagues reported the predicted number of patients with BPH-LUTS will be over 1 billion by the year 2018 [2]. The absolute prevalence rates of BPH differ in studies based several factors such as nation, longitude, and population [3, 4], but according to a study by Berry *et al*., based on biopsy and cadaver results, BPH is clearly an age-related disease [5].

Although the mechanism of prostatic enlargement is not totally clear, the relevance of androgens is considered to be one of the most important factors. Androgens may not be the direct cause of BPH, but still the testicular androgens are essential in the development of prostatic growth [6]. Among them, the dihydrotestosterone (DHT) is expected to be the most crucial factor. The serum concentration of DHT is elevated in BPH patients than that in unaffected men at the similar age [7]. In the prostate, 5α-reductase (5AR) converts testosterone into DHT [6]. DHT is a more potent androgen than testosterone because of its higher affinity for the androgen receptor (AR) [8]. Overall, almost 90% of the total prostatic androgen is in the form of DHT principally derived from testicular androgens. Inside the prostate cells, testosterone and DHT both bind to the same receptor, the AR, which results in increased transcription of androgen-dependent genes and ultimately stimulation of the protein synthesis [9]. The specific receptor, AR, is a nuclear receptor which is activated by binding of androgens in the cytoplasm and then translocate into the nucleus [10]. AR also has additional functions such as inducing the activation of kinase-signaling cascades or modulating the intracellular calcium levels [11], but in the biology of BPH, its role as a DNA-binding transcription factor which can regulate gene expression is the most important feature [12]. Expression of androgen regulated genes is affected by co-regulators that influence various functions of AR [13]. These co-regulators such as steroid receptor coactivator 1 (SRC1) modify the transcriptional activity of AR which could be related to BPH. Thus, the action of androgens in the prostate is mediated indirectly through autocrine and paracrine pathways.

BPH patients suffering from severe symptoms need to be treated with surgery or medication. Although transurethral resection of the prostate is the most common surgical treatment for BPH, this procedure can lead to negative complications, for instance bleeding, urethral stricture, and incontinence [14]. The most frequently prescribed medications are α-blockers and 5α-reductase inhibitors (5ARIs). Alpha-blocker, an antagonist against α_1_-adrenergic receptors, is a common choice for initial therapy in the USA and Europe [15–17]. They relax the smooth muscles in the prostate and the bladder neck, thus decrease the blockage of urine flow. However, they are not effective to the size of the prostate, as they cannot shrink the enlarged prostate. Furthermore, they exhibit side effects such as orthostatic hypotension, ejaculation changes, headaches, nasal congestion, and weakness [18]. On the other hand, the 5ARI is another treatment option. As its name, 5ARI inhibits the function of 5AR. The effects may take longer to appear than α-blockers, but they persist much longer, up to many years [19]. But 5ARIs also cause side effects including decreased libido, ejaculatory and erectile dysfunction [20]. Therefore, the market of new therapeutic treatments is increasing. Herbal remedies are commonly considered for the treatment of BPH [21]. USA and some European countries have approved several herbal medications, and among them, the most famous and dominant herbal therapy for BPH is Saw palmetto, which is extracted from *Serenoa repens* [22].

Chrysophanic acid (CA) is a member of the anthraquinone family. Previous studies have shown that the derivatives of anthraquinones exert a number of biological effects including anticancer [23, 24], hepatoprotective [25], antimicrobial [26], and anti-inflammatory features [27]. Even though numerous biological activities of CA have been reported, there is only limited evidence for its effect on BPH.

Since Kato *et al*. have reported increased prostate weight by treatment with testosterone propionate (TP) in 1965 [28], the rat model of BPH have been improved by Maggi and colleagues [29], currently used widely in BPH-related studies. Based on the detailed human study of McNeal [30], several homologies offer an opportunity to examine animal BPH models with the premise of understanding the mechanisms and etiology of pathological processes involved in BPH.

In this study, we demonstrated the effects of CA on BPH in TP-induced BPH rats by measuring the prostate tissue weight, examining the histological changes, and evaluating the major factors involved in the biology of BPH such as prostate specific antigen (PSA), 5AR, AR, SRC1, estrogen receptor α (ERα), and mitogen-activated protein kinases (MAPKs).

## RESULTS

### Effect of CA on the prostate tissue weight in TP-induced BPH rats

The body weights, prostate weights, and the prostate indexes of the rats are shown in Table 1. There was no significant difference in body weights of rats whether they went through TP treatment or not, or treated by CA or finasteride (Fi), the well-known 5ARI (Table 1). The TP-induced BPH group showed 1433 ± 117 mg of average prostate weight. This was significantly higher by 906 mg when compared to normal control group (527 ± 44 mg). The CA-treated group showed 656 mg decreased prostate weight (777 ± 38 mg), while the positive control group which was treated with Fi (767 ± 37 mg) showed a similar decrease by 656 mg when compared to the BPH group.

**Table 1:** Effect of CA on body and prostate tissue weights

	NC	BPH	CA	Fi
**Body weight (g)**	476.55 ± 11.33	437.00 ± 19.30	454.48 ± 22.97	466.17 ± 14.37
**Prostate Weight (mg)**	526.66 ± 43.68	1433.33 ± 116.76^#^	776.66 ± 37.52*	766.66 ± 36.85*
**Prostate index**	111.04 ± 10.75	329.60 ± 29.53^#^	171.07 ± 4.48*	165.32 ±1 1.99*

Values are mean ± S.D. of data from three separate experiments. Prostate index was calculated dividing body weight of the rat (100 g) by the prostate weight (mg). ^#^*P* < 0.05 when compared to NC; *P < 0.05 when compared to BPH. NC, normal control group; BPH, TP-induced BPH group; CA, CA-treated BPH group; Fi, Fi-treated BPH group.

**Figure 1:**
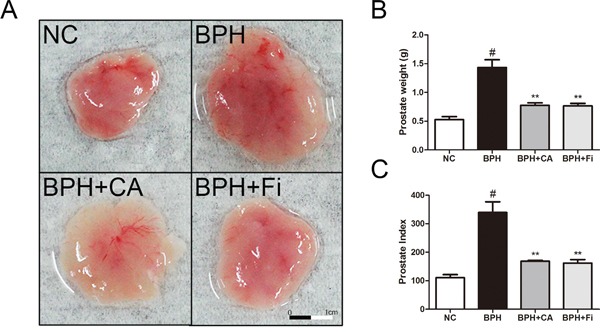
Effect of CA on prostate weight and prostate index in TP-induced BPH rats **A**. The dissection of prostates. **B**. The total prostate weight of the rats. **C**. Prostate indexes. The prostate indexes were calculated dividing prostate weight (mg) by body weight (100 g). ^#^*P* < 0.05 when compared to NC; ^*^P < 0.01 when compared to BPH. NC, normal control group; BPH, TP-induced BPH group; CA, CA-treated BPH group; Fi, Fi-treated BPH group.

Figure 1A shows visual comparisons of the prostate tissues among the four groups. The prostate weights and prostate indexes shown in Table 1. are indicated in bar graphs in Figure 1B. As shown, the BPH group had heavier prostates compared to the NC group, and the treatment of CA suppressed the prostatic growth by TP administration.

### Effect of CA on the prostate index in TP-induced BPH rats

The prostate weight index was calculated dividing total prostate tissue weight (mg) by body weight (100 g). As shown in Figure 1C, administration of TP significantly elevated the prostate weight index nearly 3 times higher than normal controlled rats (NC). Treatment with CA significantly decreased total prostate weight index when compared to TP-treated group. Similar effects were observed in Fi-treated group. The percentage inhibition was found out to be approximately 48% and 50% by CA and Fi, respectively, when compared with the TP-induced BPH group.

### Effect of CA on histological changes in TP-induced BPH rats

**Figure 2:**
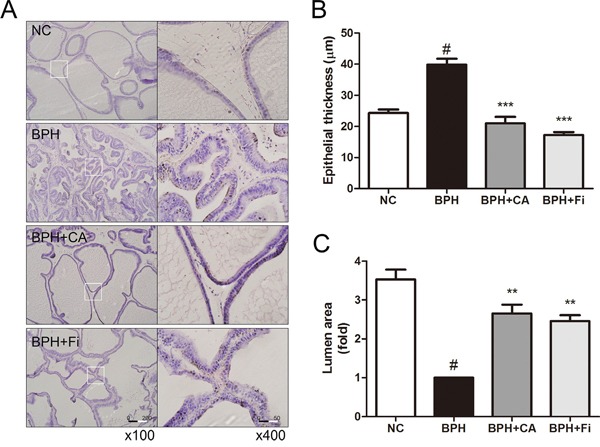
Effect of CA on histological changes of the prostate tissues in TP-induced BPH rats **A**. Representative photomicrograph of H&E stained prostate tissues (left panel magnification ×100, right panel magnification ×400). **B**. The epithelial thickness of the prostate tissues. **C**. The relative lumen area of the prostate tissues. ^#^*P* < 0.05 when compared to NC; ^*^P < 0.01 when compared to BPH; ^**^*P < 0.001 when compared to BPH. NC, normal control group; BPH, TP-induced BPH group; CA, CA-treated BPH group; Fi, Fi-treated BPH group.

To evaluate the histological changes in the prostates, an H&E staining analysis was conducted. As in Figure 2A, TP administration caused changes in the prostate structures, while CA and Fi treatment restored the histological structures similar to the NC group. In order to estimate the changes and medication effects, we have chosen 2 different features to quantify the effects.

Figure 2B shows the epithelial thickness of the prostate. TP-treated BPH group (39.9 ± 7.3 μm) produced significant increase in the epithelial thickness of the prostates by 15.6 μm than the normal rats (24.3 ± 4.4 μm). However, treatment with CA (21.0 ± 8.0 μm) significantly decreased the epithelial thickness by 18.9 μm, which was even lower than the NC group, while the Fi group (17.2 ± 3.7 μm) showed reduced thickness by 22.7 μm.

The epithelial thickness grew by TP administration as the prostatic hyperplasia occurs, in contrast, the lumen area shrunk. Administration of TP reduced the prostatic lumen area of the tissue cells lower than one-third of it of the NC group. However, treatment with CA (Figure 2C) resulted in significant increases (*p* < 0.05) in the prostatic lumen areas when compared with the BPH group. Daily treatment of Fi for 4 weeks (Figure 2C) was also capable to increase the prostatic lumen area compared to the TP-treated group.

### Effect of CA on PSA-like protein levels in TP-induced BPH rats

**Figure 3:**
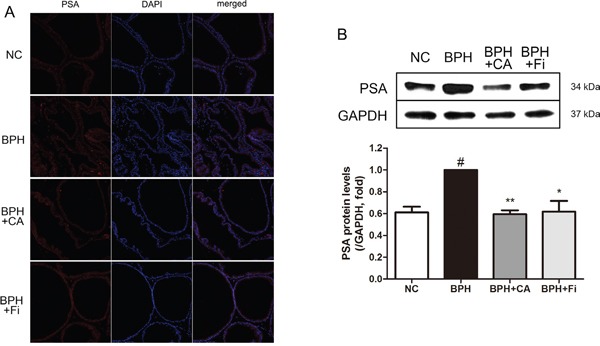
Effect of CA on PSA-like protein in the prostate tissues of TP-induced BPH rats **A**. Immunofluorescence analysis of PSA-like protein (red) and DAPI (blue) of each group. **B**. Representative western blot bands of PSA-like protein expressions of each group. **C**. The normalized relative PSA-like protein expression. The protein expressions differences were normalized to GAPDH. Values are mean ± S.D. of data three or more separate experiments. ^#^*P* < 0.05 when compared to NC; *P < 0.05 when compared to BPH; ^*^P < 0.01 when compared to BPH. NC, normal control group; BPH, TP-induced BPH group; CA, CA-treated BPH group; Fi, Fi-treated BPH group.

PSA is a unique enzyme secreted by the epithelial cells of the prostate gland. The level of PSA is often elevated in prostatic disorders [31]. PSA was formally known to be undetectable in animals besides humans [32], however, recent reports showed that anti-human PSA antibodies were able to recognize a similar PSA-like protein also in rat prostates [33, 34]. In order to evaluate the location and expression levels of this PSA-like protein to confirm whether TP injection successfully induced BPH, an immunofluorescence (IF) assay and a western blotting assay was performed. As shown in Figure 3A, TP-induced BPH rats showed highly expressed PSA-like proteins compared to normal rats, mostly localized near the nucleus. In the CA-treated group, the PSA-like protein expressions in the prostate showed similar intensity to those of the NC rats, while Fi treatment seemed to show less effectiveness. To further confirm the quantitative amount of PSA-like protein expression, a western blot analysis was carried on. As in Figure 3B, administration of TP up-regulated the PSA-like protein level nearly as twice as higher than the NC group. This elevated level of PSA-like protein was suppressed by both CA and Fi treatment.

### Effect of CA on 5AR in TP-induced BPH rats

**Figure 4:**
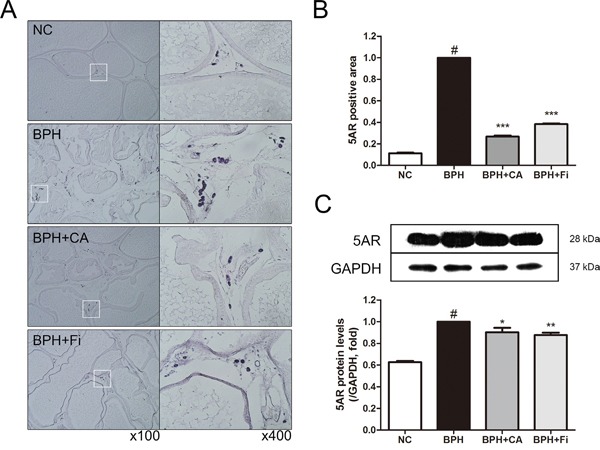
Effect of CA on 5AR in the prostate tissues of TP-induced BPH rats **A**. Representative photomicrographs of the immunohistochemically stained prostate tissues with anti-5AR antibodies (left panel magnification ×100, right panel magnification ×400). **B**. The relative densities of 5AR-positive areas. **C**. Representative western blot bands of 5AR protein expressions of each group. Values are mean ± S.D. of data from three or more separate experiments. ^#^*P* < 0.05 when compared to NC; ^**^*P < 0.001 when compared to BPH. NC, normal control group; BPH, TP-induced BPH group; CA, CA-treated BPH group; Fi, Fi-treated BPH group.

5AR is the key enzyme in the androgen function of the etiology of BPH. In order to examine the 5AR differences among the groups, an immunohistochemistry (IHC) assay and a western blot analysis was carried on. Figure 4A shows representative slides of each group in two magnifications of ×100 and ×400. As shown in Figure 4B, the stained 5AR positive area was much higher in the BPH group when compared to the NC group. The elevated staining of 5AR was down-regulated by CA or Fi treatment.

To confirm the former results in the protein level, a western blot assay was performed. As in Figure 4C, the expression of 5AR was elevated by TP treatment compared to the normal controlled group. On the other hand, treatment with CA inhibited the TP-induced 5AR elevation. The positive control Fi also suppressed the up-regulated 5AR expression.

### Effect of CA on AR and its coactivator SRC1 in TP-induced BPH rats

**Figure 5:**
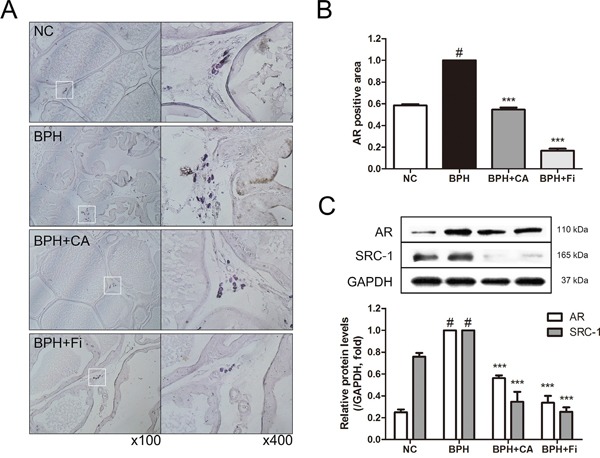
Effect of CA on AR and its coactivator SRC1 in the prostate tissues of TP-induced BPH rats **A**. Representative photomicrographs of the immunohistochemically stained prostate tissues with anti-AR antibodies (left panel magnification ×100, right panel magnification ×400). **B**. The relative densities of AR-positive areas. **C**. Representative western blot bands of AR and SRC1 protein expressions of each group. Values are mean ± S.D. of data from three or more separate experiments. ^#^*P* < 0.05 when compared to NC; ^**^*P < 0.001 when compared to BPH. NC, normal control group; BPH, TP-induced BPH group; CA, CA-treated BPH group; Fi, Fi-treated BPH group.

AR, one of the major key factors in development of BPH, was evaluated by microscopic examination of the immunostained prostate slides (Figure 5A). The TP-treated BPH group showed elevated expression levels of AR when compared with the NC group (Figure 5B). CA treatment and Fi treatment suppressed the expression of AR at the rate of 45.58 % and 84.39 %, respectively.

SRC1 interacts with AR and enhances both ligand-dependent and independent transactivation to increase transcription of androgen-regulated genes [11, 13]. The protein expression levels of AR and SRC1 were also evaluated by western blotting analyses (Figure 5C). Consist to the IHC result, the level of AR was elevated by TP administration. The CA and Fi treatment suppressed the elevated AR protein expression. Moreover, similar to the AR expressions, when the protein expressions of SRC1 were examined by a western blot assay, SRC1 expression was also up-regulated in the BPH group, and then down-regulated by CA and Fi treatment both.

### Effect of CA on ERα in TP-induced BPH rats

**Figure 6:**
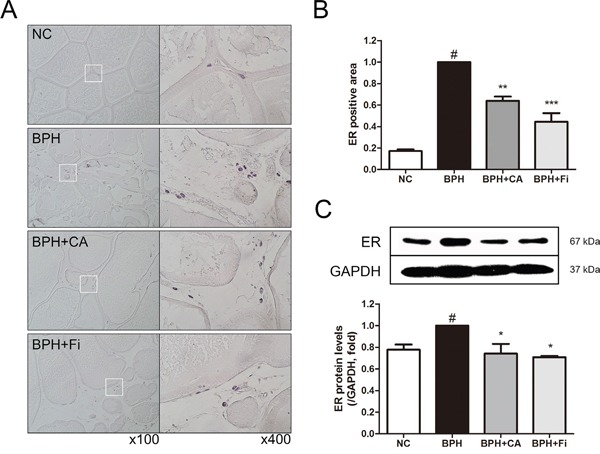
Effect of CA on ERα in the prostate tissues of TP-induced BPH rats **A**. Representative photomicrographs of the immunohistochemically stained prostate tissues with anti-ERα antibodies (left panel magnification ×100, right panel magnification ×400). **B**. The relative densities of ERα-positive areas. **C**. Representative western blot bands of ERα protein expressions of each group. Values are mean ± S.D. of data from three or more separate experiments. ^#^*P* < 0.05 when compared to NC; ^*^P < 0.01 when compared to BPH; ^**^*P < 0.001 when compared to BPH. NC, normal control group; BPH, TP-induced BPH group; CA, CA-treated BPH group; Fi, Fi-treated BPH group.

Substantial evidence has shown that stimulation of ERα and ERβ in the prostate can drive either proliferation or anti-proliferative mechanisms, respectively [35]. The immunostaining of the prostate showed elevated level of ERα in the TP-induced BPH group. However, by CA treatment, the ERα expression was decreased by 37% compared to the TP treated group, while the Fi treated group showed decreased expression of ERα by 56% (Figure 6A and 6B).

To confirm the effect of CA on ERα in the protein level, a western blot assay was carried on. As shown in Figure 6C, the protein expression of ERα was elevated in the BPH group. However, the elevated ERα was suppressed by CA treatment, consist to the IHC staining. The treatment of Fi could also suppress the elevated ERα protein expression.

### Effect of CA on MAPKs in TP-induced BPH rats

**Figure 7:**
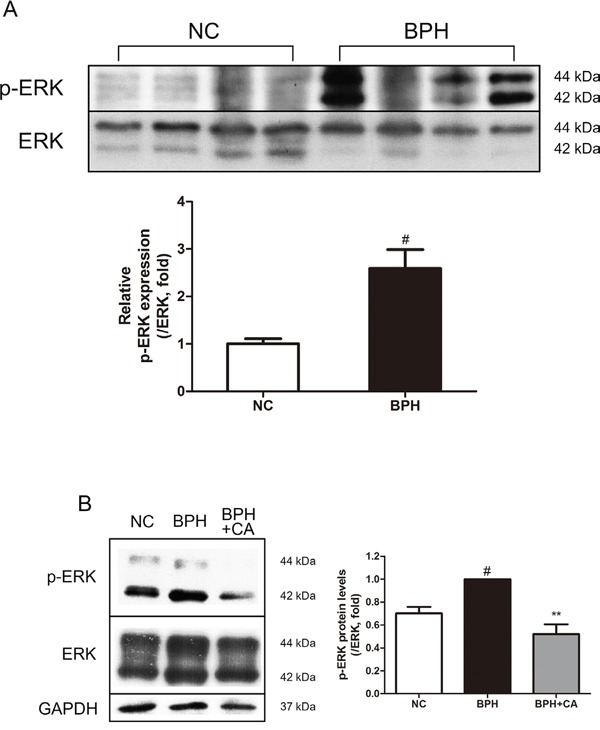
Effect of CA on ERK in the prostate tissues of TP-induced BPH rats **A**. Protein expressions of p-ERK and ERK of the NC and BPH group. **B**. Representative western blot bands of p-ERK and ERK protein expressions of each group. The protein expressions differences of p-ERK were normalized to total ERK. Values are mean ± S.D. of data from three or more separate experiments. ^#^*P* < 0.05 when compared to NC; ^*^P < 0.01 when compared to BPH. NC, normal control group; BPH, TP-induced BPH group; CA, CA-treated BPH group.

The MAPK pathway is a protein chain which is closely related to cell proliferation and apoptosis [36]. However, little is known about the MAPK signaling pathway in the etiology of BPH. We have examined the changes of 3 major kinases composing the MAPK chain; extracellular signal-regulated kinase (ERK), c-Jun-N-terminal kinase (JNK) and p38 mitogen-activated protein kinase (p38) in prostatic hyperplasia. As in Figure 7A, phosphorylation of ERK, which is known to be relevant to the cellular proliferation, was highly elevated by TP administration. On the other hand, activation of the kinase known to possess a role in apoptotic actions, JNK and p38, were not affected during the process of prostatic hyperplasia (Supplementary Figure 1A and 1B). The up-regulated expression of ERK was decreased by CA treatment similar to the levels of those from the NC group, showing the suppression of proliferation by CA (Figure 7B).

### Effect of CA on RWPE-1 prostate epithelial cells

**Figure 8:**
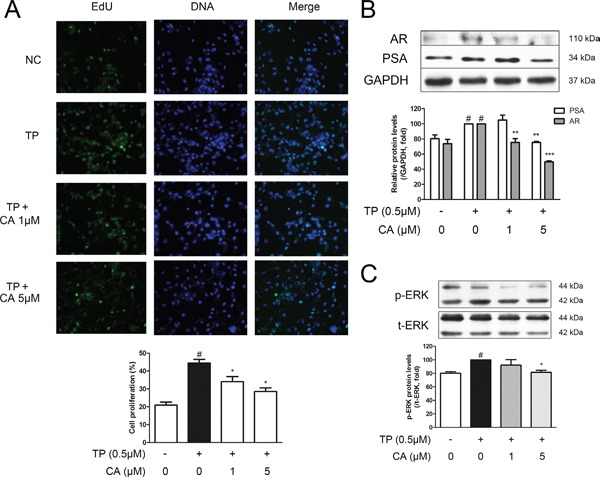
Effect of CA on proliferation and BPH-related protein expressions in RWPE-1 cells The normal human prostatic epithelial cell line, RWPE-1, were treated with 0.5 μM of TP for 24 h to induce proliferation, with or without various concentrations of CA (1 and 5 μM). **A**. Representative photomicrographs of the EdU assay. **B**. Representative western blot bands of PSA and AR protein expressions. **C**. Representative western blot bands of p-ERK and ERK protein expressions of each group. GAPDH was used as an endogenous control. The protein expressions differences of p-ERK were normalized to total ERK. ^#^*P* < 0.05 when compared to NC; *P < 0.05 when compared to TP-induced RWPE-1 cells; ^*^P < 0.01 when compared to TP-induced RWPE-1 cells.

The normal prostate epithelial cell-line, RWPE-1, was used in order to perform *in vitro* studies. An MTS assay was conducted in order to assess possible cytotoxicity of CA in RWPE-1 cells. As a result, CA did not affect the viability of RWPE-1 cells up to the concentration of 5 μM (data not shown). Next, we have established a BPH-like *in vitro* model by treating RWPE-1 cells with 0.5 μM of TP, a model which was modified from the study by Ren *et al*. [37]. By an EdU assay, we could confirm that TP treatment indeed induced proliferation of RWPE-1 cells, supporting the validity of this cellular model for BPH study. CA treatment was able to suppress this TP-induced proliferation (Figure 8A). Further western blot assays were carried out to investigate the proliferation-inhibiting mechanism of CA. By TP pre-treatment, the protein expressions of PSA and AR were elevated as in Figure 8B, and these protein increases were then suppressed in the CA-treated cells. Furthermore, the phosphorylation level of ERK was also increased by TP, supporting our ERK-related results from the *vivo* study, and 5 μM of CA successfully inhibited this increase of p-ERK (Figure 8C).

## DISCUSSION

Currently there is no completely effective treatment for BPH, despite the fact it is one of the most common diseases in elderly men [38]. The two major classes of drugs mainly prescribed to treat BPH, α-blockers and 5ARIs, show various types of side effects. Therefore, the number of patients who consider alternative medication is increasing [39], as the whole phytotherapeutic drugs exhibit remarkably benign adverse effects compared to conventional medications [22]. Besides the most widely used BPH alternative medication saw palmetto, several prescriptions or herbs from Traditional Korean Medicine are reported to be effective for BPH, such as Yukmijihwang-tang [40], *Rubus coreanus* [41], *Scutellaria baicalensis* [42], *Curcuma longa* [43], *Phellodendron amurense* [44] and *Cinnamomum verum* [45].

CA, also known as the name chrysophanol, is a rhubarb anthraquinone (C_15_H_10_O_4_) in a form of brownish-yellow powder. It is known to be abundant in the Traditional Korean Medicinal herb *Rheum palmatum* L., which is frequently prescribed for constipation. Numerous reports of CA are published, but to date, there are no reports on the effects of CA in BPH. Therefore, in this study, we investigated the effects of CA using the TP-induced BPH rat model, in order to estimate the new therapeutic potential of CA in BPH treatment. As a result, CA treatment successfully reduced the enlarged prostate tissue by TP administration (Table 1 and Figure 1). It also restored the histological changes caused by TP-induced prostatic hyperplasia (Figure 2).

PSA is a glycoprotein enzyme, which liquefies semen in the seminal coagulum to allow the sperms to swim [46]. Despite the fact it is not a unique indicator of prostate cancer, PSA is widely used to help the diagnosis prostatitis or BPH [47]. We have proved that the PSA-like protein, the protein which resembles the homolog of human PSA, was detected in the prostate tissue of rats, and the level of this PSA-like protein is elevated by TP administration, supporting the validity of TP-induced BPH rat model (Figure 3).

5AR enzymes, also known as 3-oxo-5α-steroid 4-dehydrogenases, are enzymes involved in steroid metabolism. 5ARs are known for converting testosterone into the more potent DHT. Though the mechanism of 5AR is complex, inhibition of 5AR by current BPH medications such as Fi results in decreased conversion of testosterone to DHT [48]. DHT drives its action on the prostate by binding to AR, and this binding triggers the expression of a wide array of hormone-responsive genes [11]. During this process, certain coactivators such as SRC1 are recruited to the DHT-AR bind. In the present study, CA had significant suppressive effect on 5AR, suggesting the possibility of CA as a 5ARI (Figure 4). In addition, CA has also down-regulated the level of AR and its coactivating protein SRC1 (Figure 5). As 5AR is the initial trigger of prostatic hyperplasia and AR is the main receptor related in it, these results suggest a pharmaceutical potential of CA on BPH treatment.

In addition to the down-regulation of 5AR and AR, CA treatment also suppressed the level of ERα than the BPH group, which may suggest another potential action mechanism of CA besides the 5AR-AR axis. Whereas the role of androgens in BPH has been extensively studied for decades, the effect of estrogens has only recently gained attention. Since the early speculations about the potential role of ERs in prostate biology [49], the effects of estrogens in BPH are recently reviewed, and gaining renewed interest. Different expression patterns and localizations of ERα and ERβ in the prostate make them able to activate or repress the growth control pathways [35]. An increased expression of ERα with the concomitant decrease of ERβ has been shown to correlate with BPH and other prostate-proliferating diseases [50]. In this study as well, we investigated the increased changes of ERα in BPH and demonstrated that CA down-regulates the ERα expression (Figure 6).

The MAPK pathway is considered very important in the cellular signaling, especially in the proliferation and apoptosis of the cells [36]. The roles of the MAPKs are well reported in prostate cancer [51, 52], but on the other hand, only limited literature are dealing this signaling chain in BPH. Papatsoris and Papavassiliou have suggested the action of MAPK families in the pathogenesis in BPH in 2001 [53]. They proposed a hypothetical model of BPH with ERK, JNK, and p38 signaling; activated ERK induces proliferation, while JNK and p38 are inhibited and therefore apoptosis is suppressed. As expected, our results showed elevated p-ERK expression by TP treatment, and this increase of phosphorylation was suppressed by CA. However, conflicting to the suggested role of JNK and p38, our results indicated these certain kinases were not related in the process of TP-induced BPH (Supplementary Figure 1). Our results show that CA treatment was able to decrease the elevated levels of p-ERK (Figure 7), but still, the exact mechanism and roles of MAPKs in BPH requires further investigation.

RWPE-1 cells are epithelial cells derived from a normal adult human prostate, responsive to androgen [54]. Our previous study reported RWPE-1 cells successfully expressed PSA and AR [45]. To establish a cellular environment similar to BPH, we treated RWPE-1 cells with TP and observed proliferation and increased levels of PSA, AR and p-ERK, which were all suppressed by CA treatment (Figure 8).

In this present study, we have shown that CA has suppressive effects on the TP-induced prostatic enlargement in rats. The weight of the prostate tissue was increased by TP injection while CA treatment reduced it. The histological changes by TP treatment, such as epithelial thickness and lumen area, were also restored by CA like those of the normal prostate group. The expression of PSA-like protein was altered by TP administration, and the elevated PSA-like protein was suppressed by CA treatment. In addition, TP administration induced the elevation of functionally central enzyme of BPH, 5AR, but CA treatment down-regulated the expression rate. The elevated BPH-related factors, AR, ERα, and the AR-coactivating protein SRC1 were also inhibited by CA treatment. The level of p-ERK, an important MAPK member related to cell proliferation which was also up-regulated in the BPH group, was reduced similar to normal by CA. Furthermore, *in vitro* studies regarding RWPE-1 prostate epithelial cells showed that CA can suppress TP-induced proliferation and TP-induced increases of PSA, AR and p-ERK. Thus, the beneficial effects of CA on prostate hyperplasia were demonstrated in this study. Overall, the results of this study indicate a potential of CA in a new pharmacotherapy treatment for BPH.

## MATERIALS AND METHODS

### Chemical reagents

CA (≥ 98 % pure) was purchased from Sigma Chemicals (St. Louis, MO, USA) and was dissolved in 100% dimethyl sulfoxide (DMSO). TP was provided from Wako pure chemical industries (Osaka, Japan), and Fi (≥ 97 % pure) was purchased from Sigma-Aldrich Inc. (St. Louis, MO, USA). Antibodies for AR, ERα and SRC1 were purchased from Pierce biotechnology (Rockford, IL, USA), antibody for 5AR was a product from Abcam Inc. (Cambridge, MA, USA). The Antibodies for PSA, p38, and Glyceraldehyde-3-phosphate dehydrogenase (GAPDH) was from Santa Cruz Biotechnology (Santa Cruz, CA, USA). p-p38, p-ERK, p-JNK, and JNK were purchased from Cell Signaling Technology (Danvers, MA, USA). Anti-ERK antibody was obtained from Bioworld Technology (St. louis Park, MN, USA).

### Animals

12-week-old male Sprague-Dawley (SD) rats (*n* = 28) with initial body weights of 200-220g were purchased from the Dae-Han Experimental Animal Center (Dae-Han Biolink, Eumsung, Korea). The animals were all maintained in conditions in accordance with the regulation issued by the Institutional Review Board of Kyung Hee University (confirmation number: KHUASP(SE)-P-034). The rats were housed in a pathogen-free room maintained at 23 ± 2°C and a relative humidity of 70% with an alternating 12 h light/dark cycle. Water and standard laboratory diet (CJ Feed Co., Ltd., Seoul, Korea) were provided *ad libitum*.

### Experimental procedures

BPH was induced by a pre-4-week treatment of daily subcutaneous injections of TP (5 mg/kg) at the inguinal region (*n* = 28) as described previously [45]. In order to set the normal control group, 7 rats received ethanol with corn oil instead of TP. After the pre-treatment of 4 weeks, the BPH induced rats were randomly divided into three groups, and the rats those did not receive TP treatment became the normal control group. Thus, the rats were divided into four groups with 7 animals in each group: (a) a normal control group that received ethanol with corn oil; (b) a BPH group that received TP with corn oil; (c) a positive control group that received finasteride (Fi) (1 mg/kg), a 5ARI which is frequently used as a treatment for BPH [20], with TP (5 mg/kg); and (d) a group that received CA (5 mg/kg) with TP (5 mg/kg). CA and Fi were administered by injection into the inguinal area of animals once daily for 4 weeks, following the pre-4-week BPH inducement. After the final treatment, animals were fasted overnight and euthanized using CO_2_. Blood samples were obtained from the caudal vena cava. The blood containing tubes were remained at RT for 2 h and sera were separated by centrifuging at 3000 × *g* for 20 min in 4°C. The serum was stored at -80°C until further assays. The intact prostate tissue was carefully dissociated and removed, washed with PBS, and then weighed. Relative prostate weight was calculated as the ratio of prostate weight (mg) to body weight (100 g). The prostate tissue was divided in half; one half was fixed in 10% formalin and embedded in paraffin for histomorphological assays, the other was stored at -80°C for further assays.

### Hematoxylin and eosin (H&E) staining and immunohistochemistry (IHC)

The prostate tissue sections were prepared as described previously [45]. For H&E staining, the sections were stained in hematoxylin for 5 min, and then washed with water for 5 min. Then the sections were stained in eosin for 30 s, dehydrated, and mounted by routine methods. For immunostaining, sections were incubated in 4°C overnight with a 1:50 dilution of the primary antibody; then incubated at room temperature for 30 min with a 1:500 dilution of the horseradish peroxidase (HRP)-conjugated affinipure Goat anti-rabbit IgG (Jackson Immunoresearch lab., West Grove, PA, USA) or HRP-conjugated affinipure Goat anti-mouse IgG (Jackson Immunoresearch lab., West Grove, PA, USA). Following the addition of the detection system, the reaction was visualized using diaminobenzidine (DAB) in the presence of hydrogen peroxide. The slides were examined using the Olympus IX71 Research Inverted Phase microscope (Olympus Co., Tokyo, Japan), and the density was measured using ImageJ 1.47v software (National Institute of Health, Bethesda, MD, USA).

### Western blotting assay

Western blotting assays were performed as described previously [45]. Briefly, Homogenized tissues or harvested cells were lysed with ice-cold RIPA buffer, the insoluble materials were removed, and the proteins were separated by 8% sodium dodecyl sulfate-polyacrylamide gel electrophoresis and transferred onto Polyvinylidenedifluoride (PVDF) membranes (Billerica, MA, USA). After blocking with 10 mM Tris, 150 mM NaCl, and 0.05 % Tween-20 (TBST) containing 5% skim milk for 1 hour at room temperature, the membranes were incubated with the primary antibody at 4°C overnight. The blots were subsequently incubated with HRP-conjugated affinipure Goat anti-rabbit IgG (Jackson Immunoresearch lab.) or HRP-conjugated affinipure Goat anti-mouse IgG (Jackson Immunoresearch lab.). The protein assay reagent was obtained from Bio-Rad (Hercules, CA, USA). The chemiluminescent intensities of protein signals were quantified using ImageJ 1.47v software (National Institute of Health).

### Cell culture

The normal human prostatic epithelial cell line RWPE-1 was obtained from the American Type Culture Collection (Manassas, VA, USA). RWPE-1 cells were cultured in Roswell Park Memorial Institute medium (RPMI) (Gibco, Big Cabin, OK, USA) supplemented with 100 mg/ml penicillin/streptomycin (HyClone, Logan, UT, USA) and 10% FBS (Sigma-Aldrich Inc.). After 24 h of incubation, the culture media was replaced with fresh media containing 0.5 μM of TP in order to induce cell proliferation. CA was supplemented together within TP-containing media.

### MTS assay

RWPE-1 cells were seeded (3 × 10^4^ cell/well) and incubated in RPMI plus 10% FBS for 24 h. Then the cells were incubated in fresh media containing various concentrations of CA for an additional 24 h. Cell viability was monitored using the cell proliferation MTS kit by the Promega Corporation as recommended by the manufacturer. Prior to measuring the viability, the media were removed and replaced with 200 μl of fresh RPMI plus 10% FBS medium and 10 μl of 3-(4,5-dimethylthiazol-2-yl)-5-(3-carboxymethoxyphenyl)-2-(4-sulfophenyl)-2*H*-tetrazolium (MTS) solution. The cells were then incubated in the incubator for 4 h. The absorbance was measured at 490 nm in a VERSAmax microplate reader (Molecular Devices, Sunnyvale, CA, USA) to determine the formazan concentration, which is proportional to the number of live cells.

### EdU proliferation assay

Proliferation of RWPE-1 cells was assessed using the Click-iT EdU Imaging Kit (Invitrogen, Waltham, MA, USA) according to the manufacturer´s protocol. Briefly, cells were plated overnight and labeled with 10 μM EdU for 24 h, and then the cells were transferred into a 6-well plate and fixed with 3.7% formaldehyde for 15 min at RT, washed twice with 3% BSA in PBS. Next, 1 ml of 0.5% Triton X-100 in PBS was added, followed by a 20 min incubation at RT. EdU was detected by staining cells with Click-iT reaction cocktail for 30 min at RT. DNA staining was performed using Alexa Fluor 488 Imaging Kit (Invitrogen, Waltham, MA, USA). Samples were analyzed using iRiS Digital Cell Imaging System (Logos Biosystems, Anyang, Korea) at 488 nm.

### Statistical analysis

The data values were presented as the mean ± standard deviation (S.D.). Differences in mean values were analyzed by one-tailed Student's *t*-test using IBM SPSS Statistics 22 software (International Business Machines Corp., New York, NY, USA). Values with *P* < 0.05 were considered as statistical significance.

## SUPPLEMENTARY FIGURE


