# Supplementary material for: Chrysophanic acid reduces testosterone-induced benign prostatic hyperplasia in rats by suppressing 5α-reductase and extracellular signal-regulated kinase
Source: Oncotarget. 2016 Nov 17;8(6):9500–12. doi: 10.18632/oncotarget.13430 (PMC5354748; doi:10.18632/oncotarget.13430)
Supplement: Supplementary file 1 [file oncotarget-08-9500-s001.pdf]

Chrysophanic acid reduces testosterone-induced benign prostatic hyperplasia in rats by suppressing 5α-reductase and extracellular signal-regulated kinase

SUPPLEMENTARY FIGURE

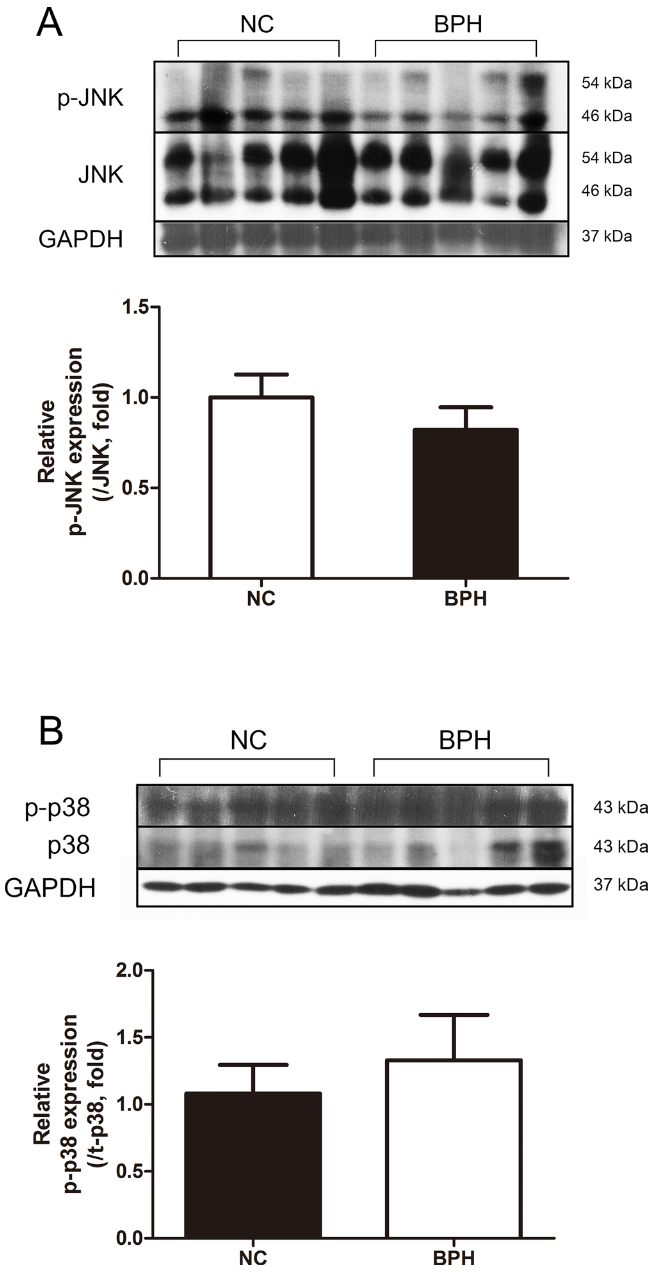

Supplementary Figure 1: Effect of CA on JNK and p38 in the prostate tissues of TP-induced BPH rats. A. Protein expressions of p-JNK and JNK of the NC and BPH group. **B.** Protein expressions of p-p38 and p38 of the NC and BPH group. The protein expressions differences of p-JNK and p-p38 were normalized to total JNK and total p38, respectively. Values are mean ± S.D. of data from three or more separate experiments. NC, normal control group; BPH, TP-induced BPH group.
